# Supplementary material for: Low abundance members of the gut microbiome exhibit high immunogenicity
Source: Gut Microbes. 2022 Jul 26;14(1):2104086. doi: 10.1080/19490976.2022.2104086 (PMC9331198; doi:10.1080/19490976.2022.2104086)
Supplement: Supplemental Material [file KGMI_A_2104086_SM7304.docx]

**Supplementary Information**


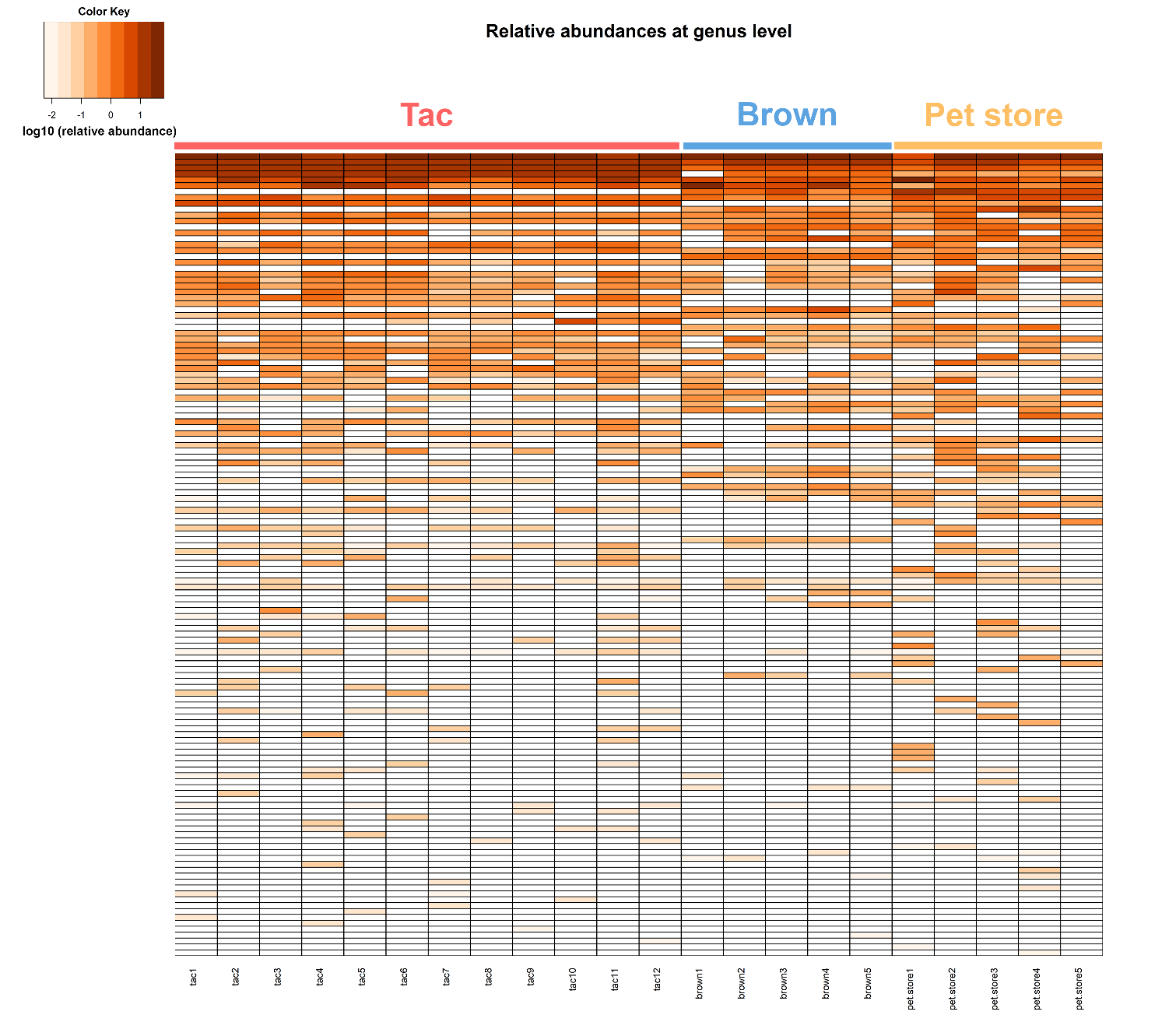


**Fig. S1. Heat map of relative abundance at the genus level of the gut microbiome of Taconic, Brown, and pet store mice.**


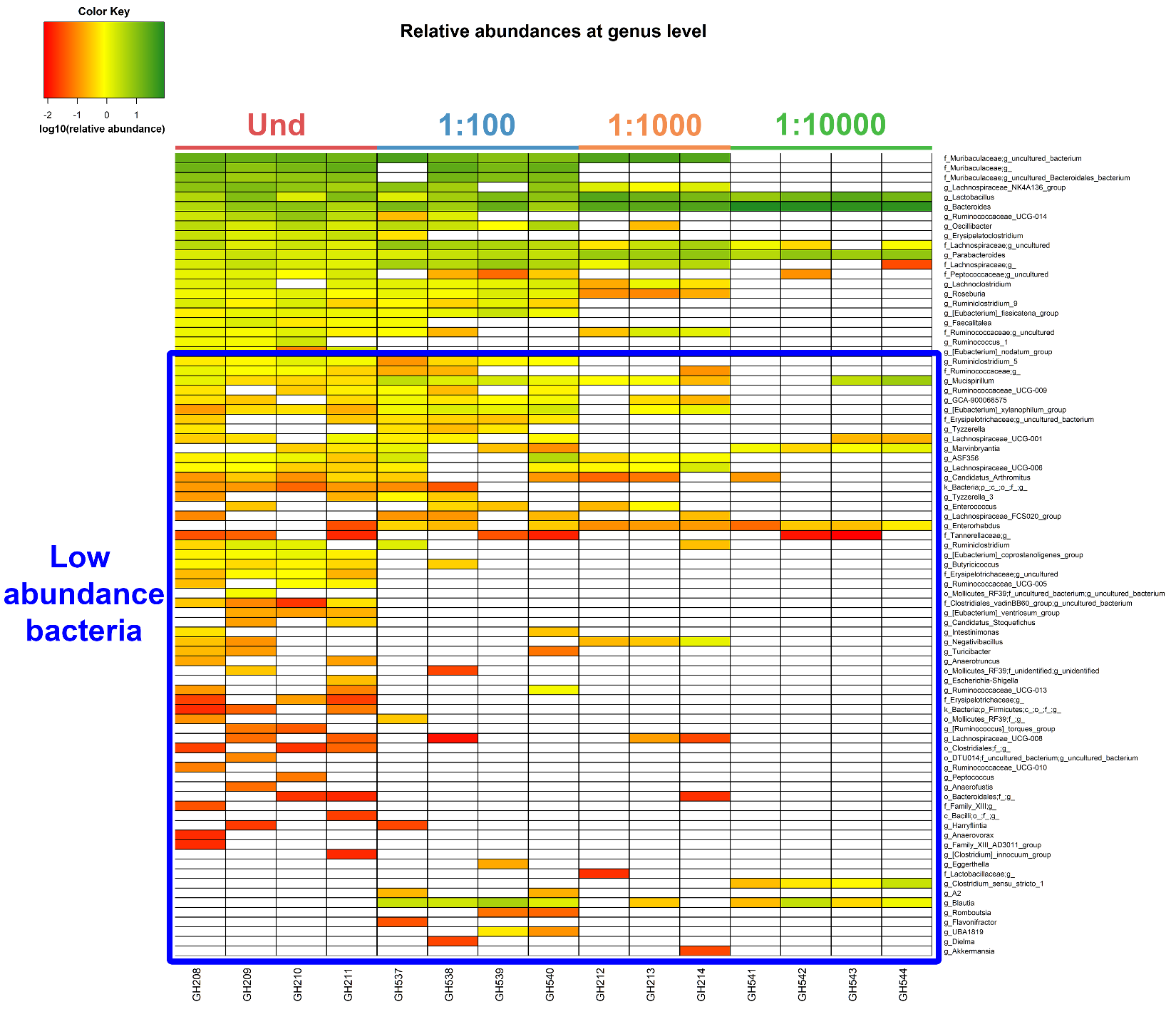

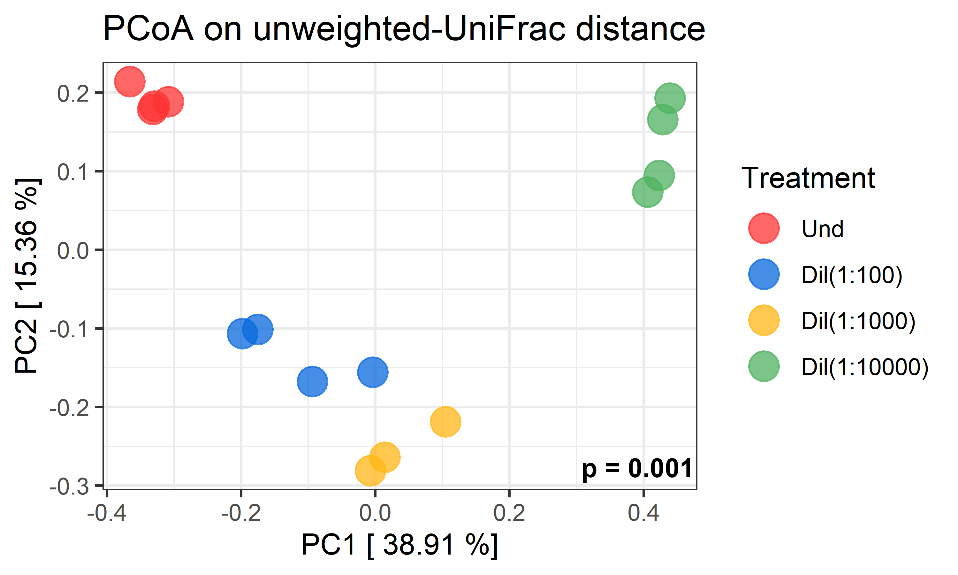


**A**

**B**

**Fig. S2. Composition of the colon microbiome in germ-free mice colonized with diluted cecal contents at various levels of dilution.** (A) PCoA plot based on unweighted-UniFrac distance. (B) Heat map of the relative abundance at the genus level. Blue box represents low abundance bacteria of the undiluted cecal contents. A relative abundance of the genus is expressed as log10(relative abundance).

**A**


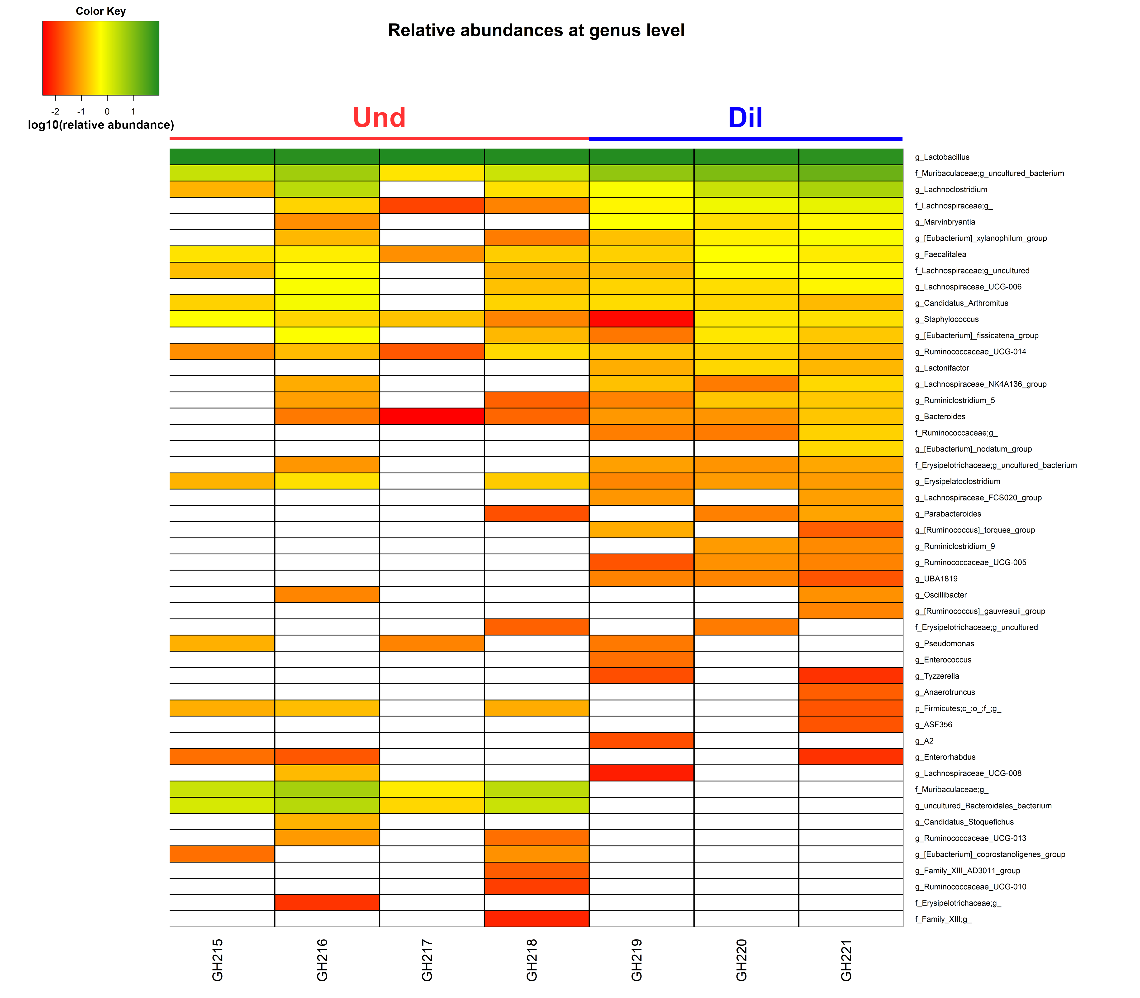

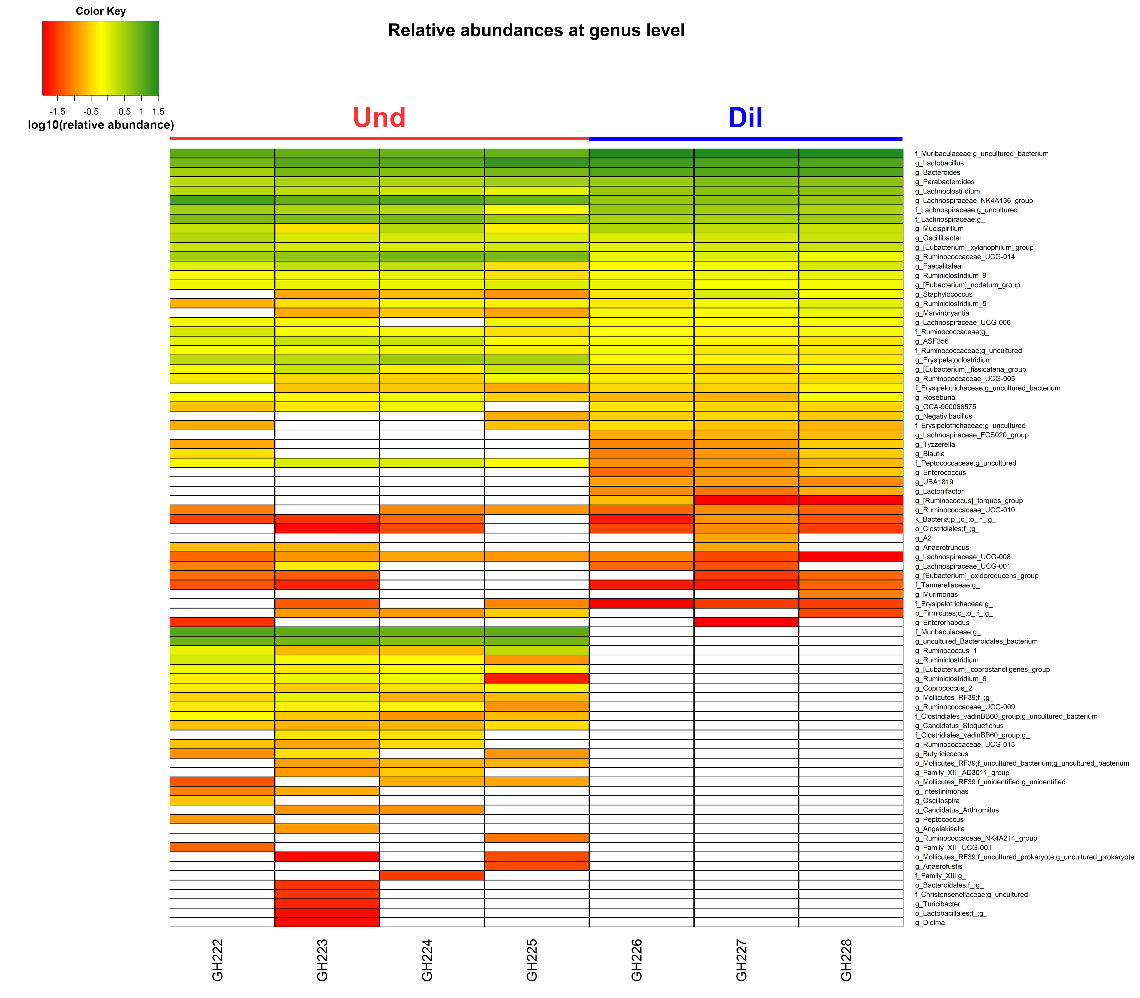


**B**

**Fig. S3. Heat map of the colon and small intestine microbiome at the genus level after five weeks of colonization with the undiluted or diluted microbiome in germ-free mice.** (A) Colon. (B) Small intestine. A relative abundance of the genus is expressed as log10(relative abundance).

**A**

**C**

**B**

**D**


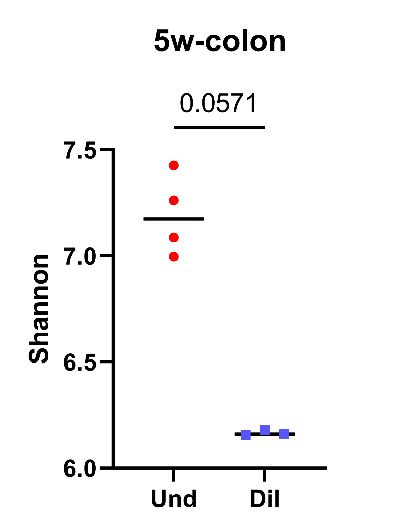

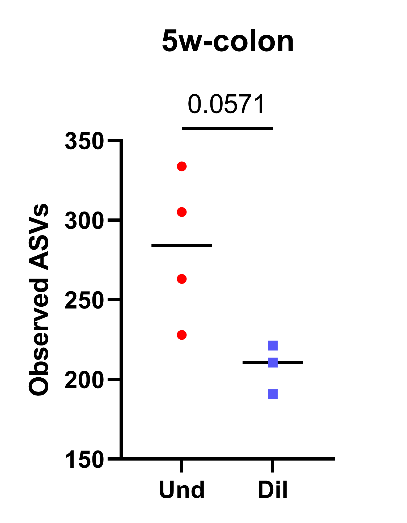

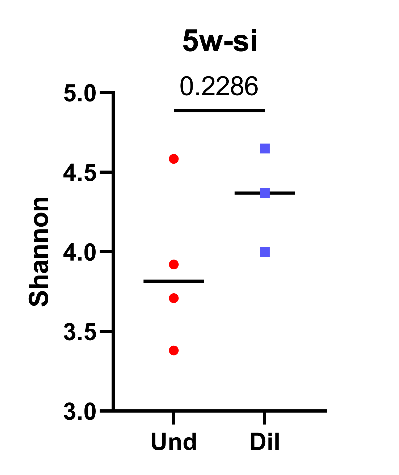

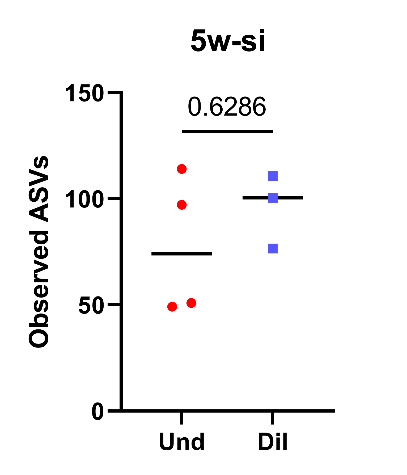

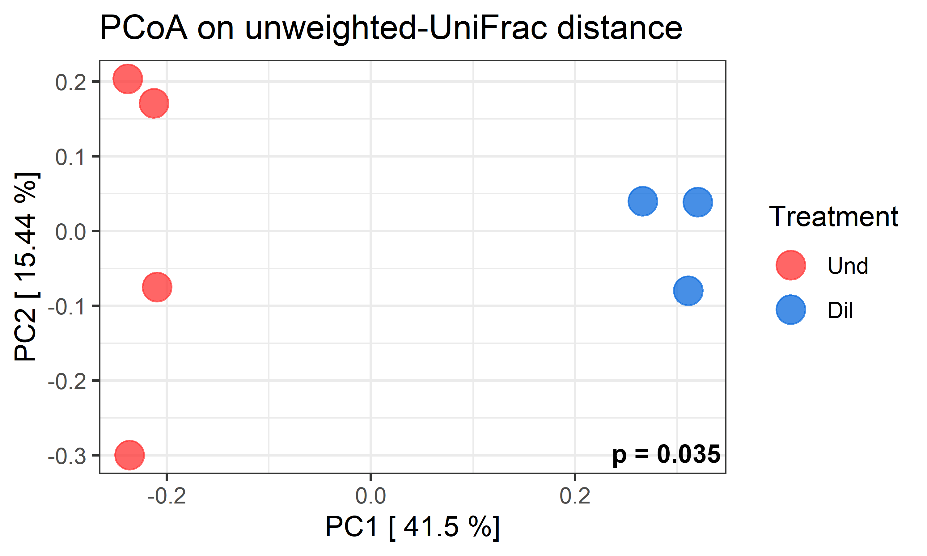

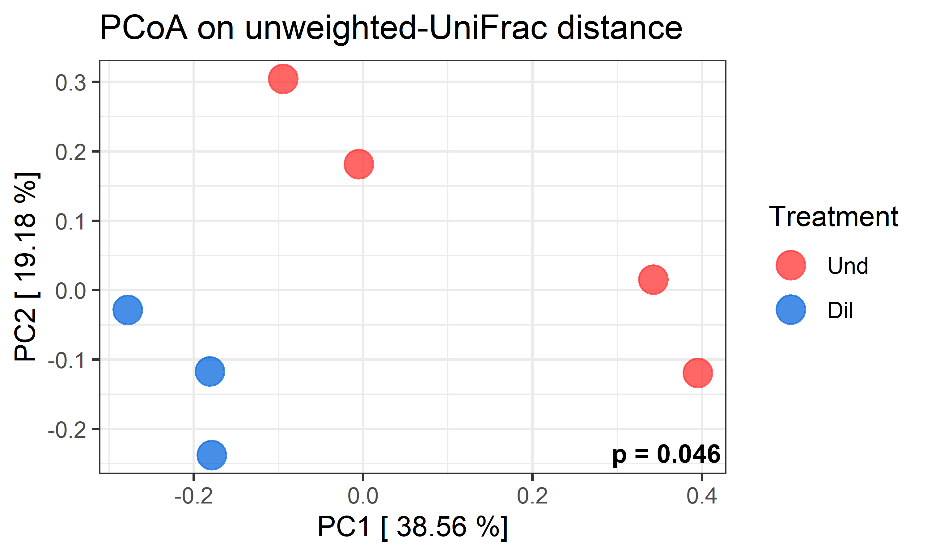


**Fig. S4. Alpha and beta diversity of the Und and Dil group in the colon and small intestine at 5-weeks post-colonization.** (A) Alpha diversity (observed ASVs and Shannon) in the colon. (B) PCoA plot based on unweighted-UniFrac distance in the colon. (C) Alpha diversity (observed ASVs and Shannon) in the small intestine. (D) PCoA plot based on unweighted-UniFrac distance in the small intestine. Mann-Whitney test and PERMANOVA were used to assess significant differences for alpha and beta diversity, respectively**.**

**
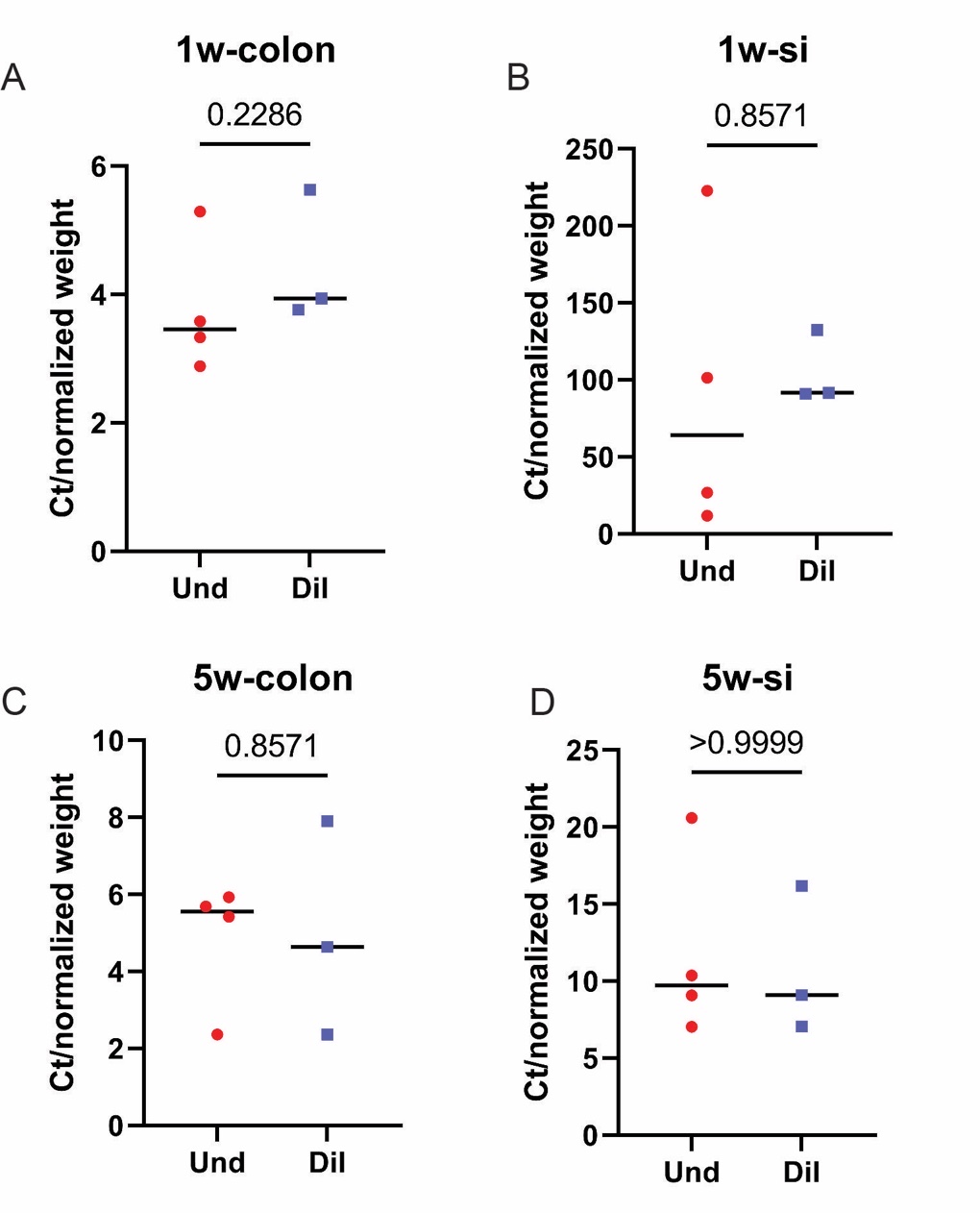
**

**Fig. S5. Bacterial load in the colon and small intestine at 1- and 5-weeks post-colonization.** Bacterial load in the colon (A) and small intestine (B) at 1-week post-colonization. Bacterial load in the colon (C) and small intestine (D) at 5-weeks post-colonization. Mann-Whitney test was used to assess significant differences between the two groups.


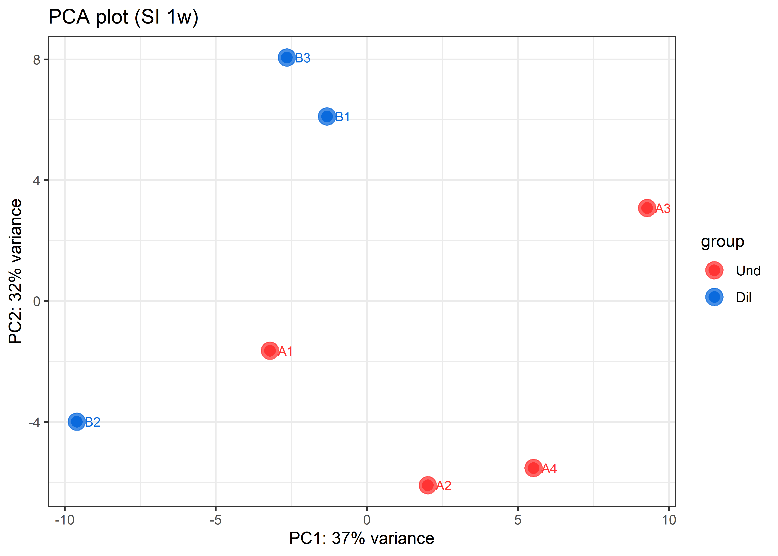

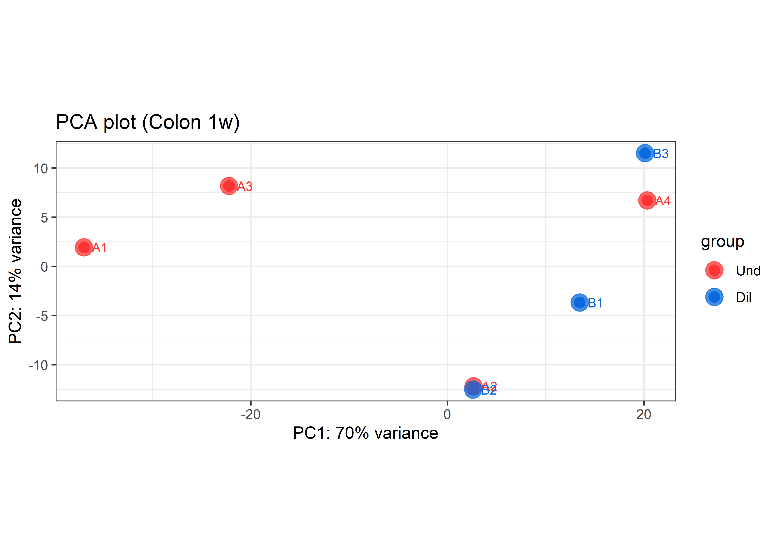


**A**

**C**

**B**

**D**


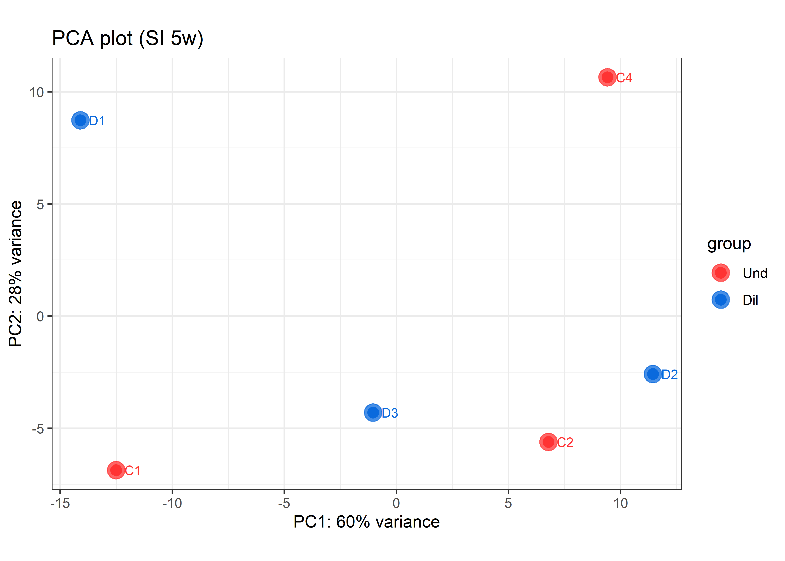

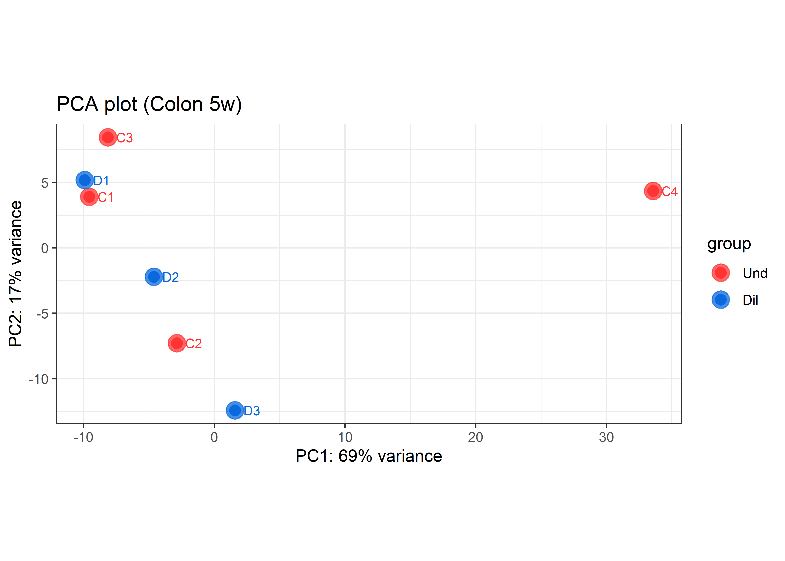


**Fig. S6. Global gene expression pattern based on RNA-Seq results.** PCA plot in the colon

(A) and small intestine (B) at 1-week post-colonization. PCA plot in the colon (C) and small

intestine (D) at 5-weeks post-colonization.


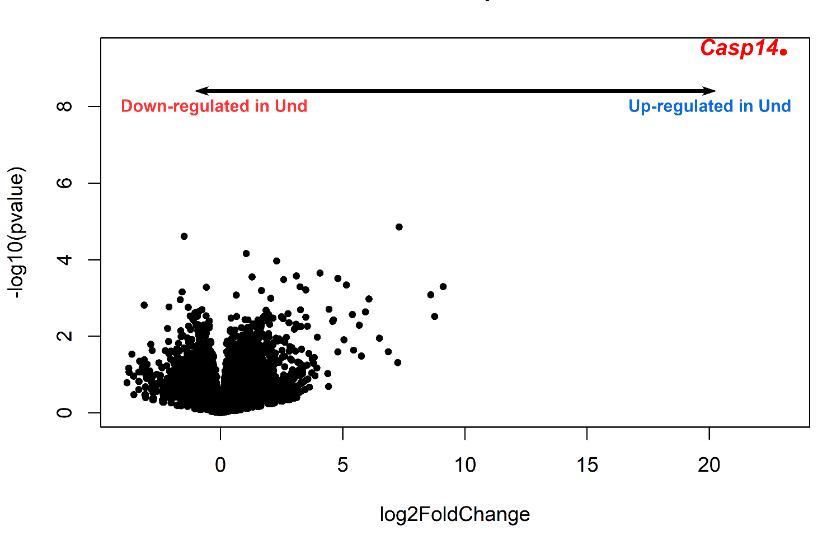


**Fig. S7. Volcano plot in the colon at 1-week post-colonization.** Each dot represents each gene, and significant DEGs are expressed as blue (FDR < 0.05) and red dots (log2FC>2, FDR < 0.05).


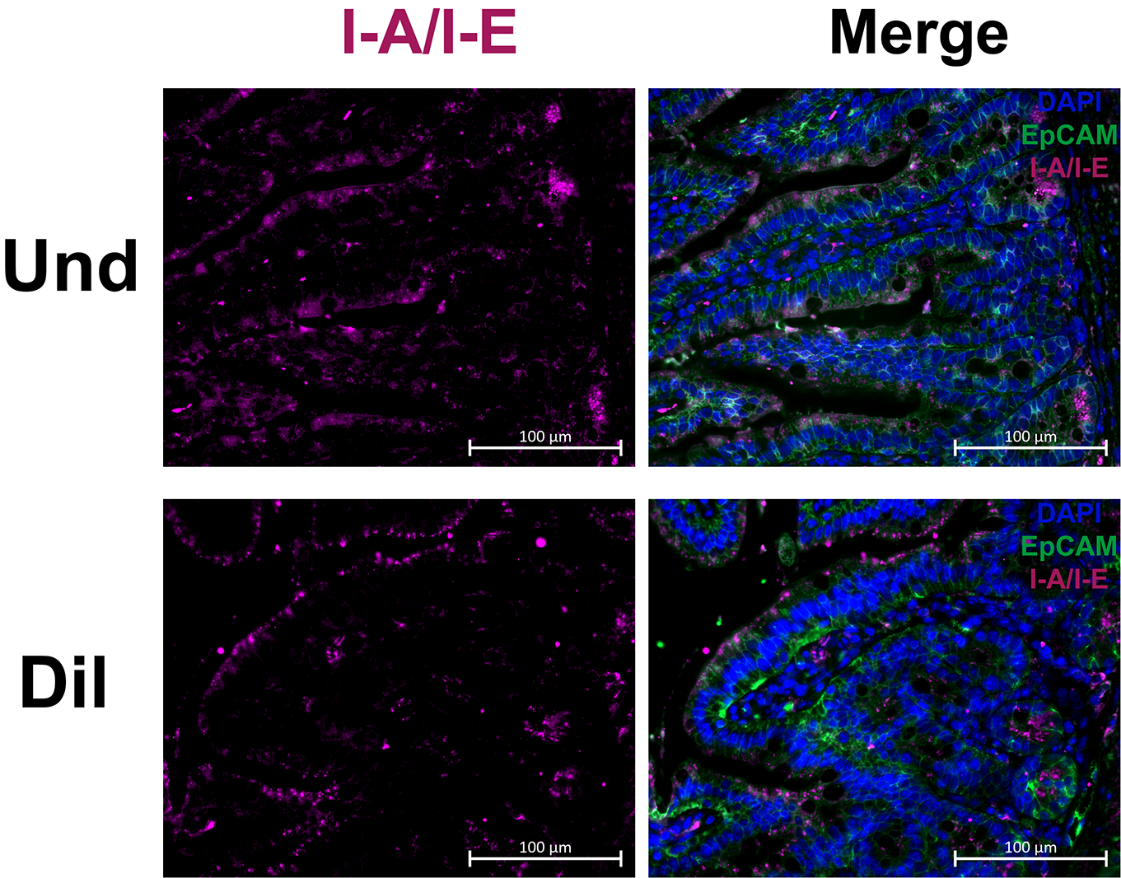

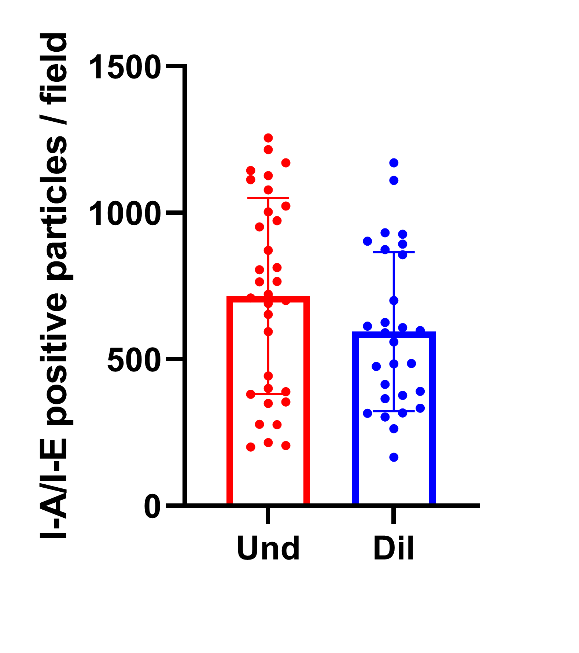


**Fig. S8. Representative images and quantification of MHC class II expression in the small**

**intestine of the Und and Dil at 5-weeks post-colonization.** Samples were stained with DAPI(nuclei; blue), EpCAM (epithelial cells; green), and I-A/I-E (MHC class II; violet). For quantification of MHC class II molecules, 6-10 images were used per mouse with 3-4 mice per group. Welch’s t-test was used to find significant differences between the two groups.


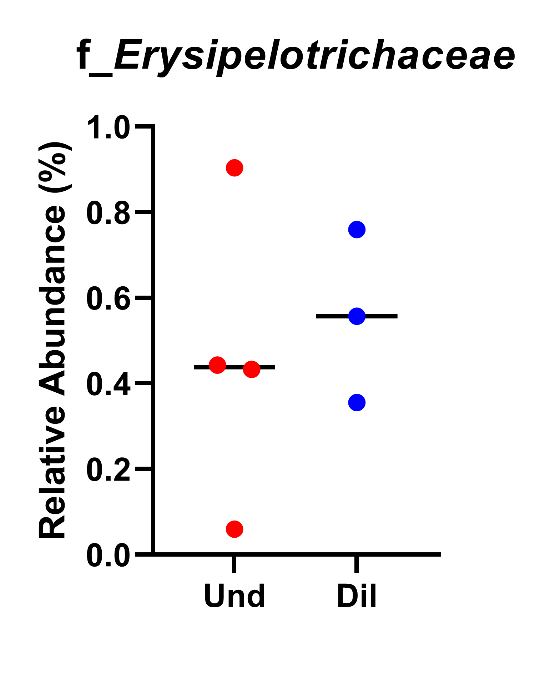

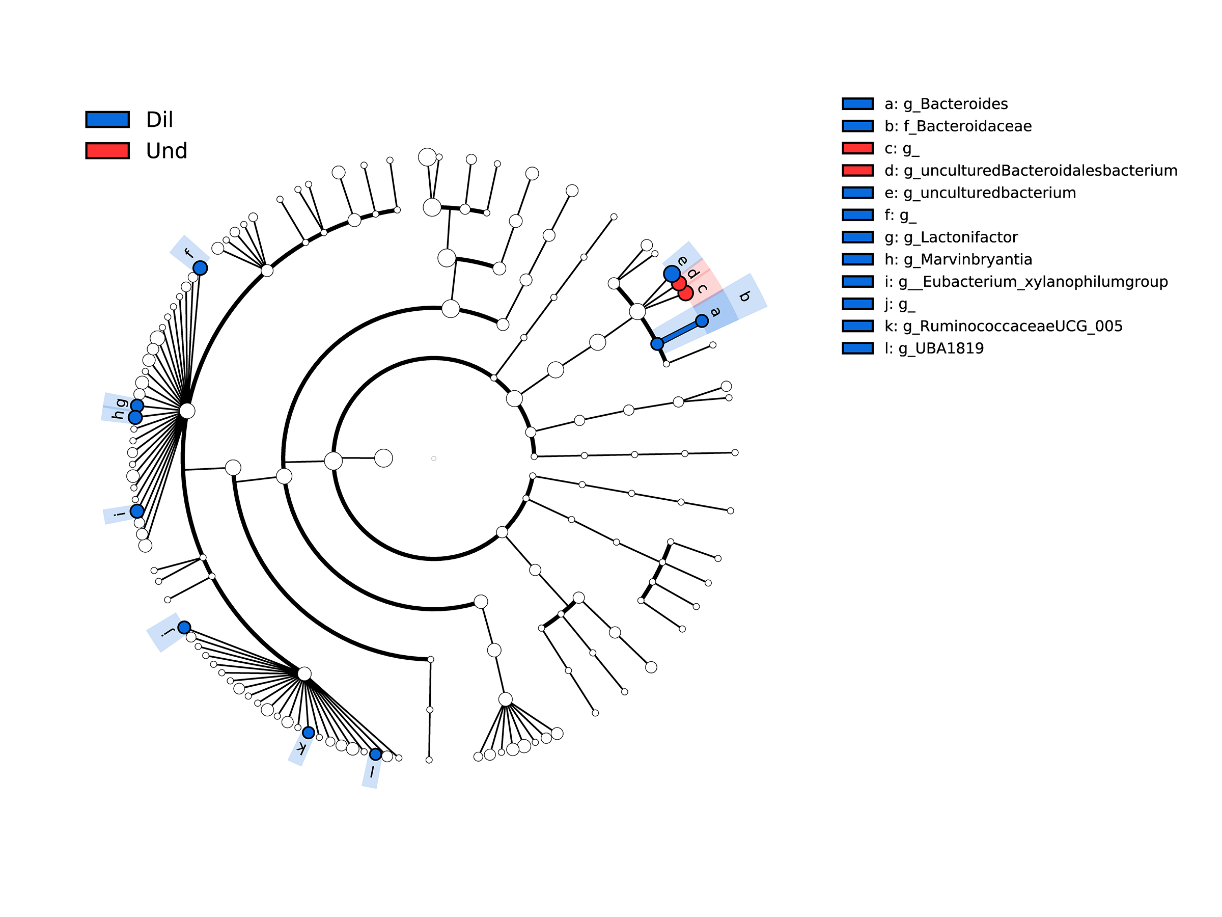

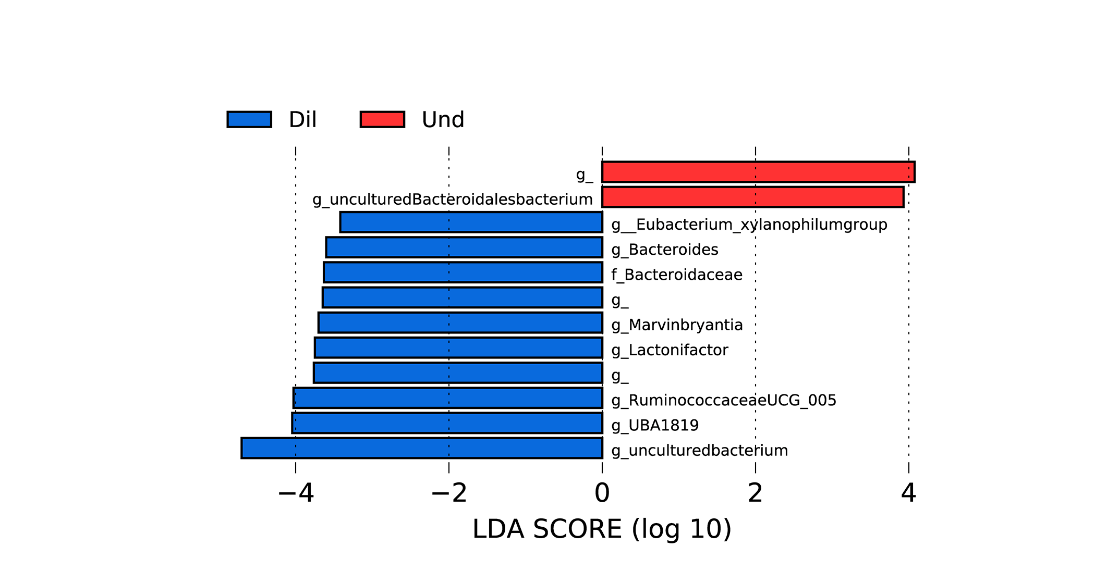


**A**

**B**

**C**

**Fig. S9. Differentially abundant taxa between the Und and Dil in the small intestine at 5**

**weeks post-colonization.** Cladogram (A) and bar plot (B), which were obtained from LEfSe

analysis, show differentially present taxa between the Und and Dil. (C) Relative abundance of

*Erysipelotrichaceae*.
